# Cannabis and cancer: unveiling the potential of a green ally in breast, colorectal, and prostate cancer

**DOI:** 10.1186/s42238-024-00233-z

**Published:** 2024-05-16

**Authors:** Husam A. ALSalamat, Sara Feras Abuarab, Hazem Mohamed Salamah, Anas Hasan Ishqair, Mohammad Fuad Dwikat, Anas Zakarya Nourelden, Aseel N. Qandil, Yasmeen Barakat, Muna Barakat

**Affiliations:** 1 Department of Basic Medical Sciences, Faculty of Medicine, Al-Balqa Applied University, Al-Salt, 19117 Jordan; 2 Department of Biopharmaceutics and Clinical Pharmacy, School of Pharmacy,, University of Jordan, Amman, 19328 Jordan; 3 International Medical Research Association (IMedRA), Cairo, Egypt; 4 Department of Clinical Pharmacy and Therapeutics, School of Pharmacy, Applied Science Private University, Amman, 541350 Jordan; 5 School of Medicine, Zagazig University, Zagazig, 44519 Egypt; 6 Faculty of Medicine, The Hashemite University, Zarqa, 13133 Jordan; 7 Faculty of Medicine, An-Najah National University, Nablus, Palestine; 8 Faculty of Medicine, Al-Azhar University, Cairo, Egypt

**Keywords:** Cannabis, Breast, Colorectal, Prostate, Cancer, Cannabinoids, Anti-cancer

## Abstract

Cancer comes in second place on the list of causes of death worldwide. In 2018, the 5-year prevalence of breast cancer (BC), prostate cancer (PC), and colorectal cancer (CRC) were 30%, 12.3%, and 10.9%, respectively. Cannabinoids are chemicals derived from the *Cannabis sativa* plant; the most investigated cannabinoids are cannabinol, delta 9-tetrahydrocannabinol (Δ^9^-THC), and cannabidiol. In humans, the endogenous endocannabinoid system consists of endocannabinoids, cannabinoids receptors (CBs), and enzymes that degrade the endocannabinoids. In this review, we will review the most recent literature for evidence that discusses the role of cannabis in the treatment of the three types of neoplasms mentioned. Studies have proved that BC cells express CB receptors; many in-*vivo* studies showed that cannabinoids cause apoptosis and inhibit proliferation and migration. Also, researchers found that treating BC mice with THC and JWH-133 (CB2 receptor agonist) slowed the tumor growth. Regarding CRC, cannabidiol was found to decrease the viability of chemotherapy-resistant CRC cells and inhibit metastasis by antagonizing the G-protein-coupled receptor 55 (GPR55; a novel cannabinoid receptor) necessary for metastasis. Moreover, cannabidiol had anti-angiogenetic effects by reducing the expression of vascular endothelial growth factor (VEGF) in addition to anti-inflammatory effects. Finally, studies demonstrated that PC cells highly express CB1 and CB2 receptors and that cannabinoids are capable of inhibiting the release of exosomes and microvesicles related to cancer progression. Cannabinoids also have antiproliferative, anti-invasive, anti-fibroblastic, cell cycle arrest, and proapoptotic effects on PC cells.

## Introduction

Cancer is the second most common cause of death worldwide. In 2018, 9.6 million deaths were attributed to cancer while 18 million new cancer cases were diagnosed. By 2030, deaths and new cases are expected to increase by 37.9% and 36.3%, respectively. Breast (30%) and prostate (17.7%) cancers (BC and PC) are the most prevalent in females and males, respectively, while colorectal cancer (CRC) is the second most prevalent cancer in both sexes (Ferlay et al. 2019).

In 2018, over 600,000 women and 350,000 men died due to BC (number one cancer killer in females) and PC (number five cancer killer in males), respectively. In the same year, BC was the most occurring among women with an incidence of over two million women diagnosed, and PC was the second most occurring in men with an incidence of 1.2 million new cases. In the same year, the 5-year prevalence of BC was approximately 30% of all female cancer patients and the 5-year prevalence of PC was 12.3% among males. CRC was the fourth leading cause of cancer-related deaths in males, the 3^rd^ in females, and the 2^nd^ in both sexes. Also, CRC had an incidence of 1.8 million new cases making it the 3^rd^ most occurring in both sexes with a 5-year prevalence of 10.9% (Ferlay et al. 2019).

The most evident risk factors of BC include obesity, using combined estrogen and progestin hormones after menopause, alcohol consumption, early menarche, late menopause, family history of BC, and genetic predisposition especially BRCA1 and BRCA2 mutations (Friedman et al. 2006, Smith-Warner et al. 1998, Tamimi et al. 2016). The most clinically evident complication of BC is metastasis and the most common site is the bone (Kennecke et al. 2010, Xiao et al. 2018). For PC, the most evident and researched risk factors are age which has a direct relationship with the risk of developing PC, ethnicity (making the highest risk among African Americans), in addition to family history (Cuzick et al. 2014, Markozannes et al. 2016). PC has a very high chance of metastasis compared with other neoplasms, especially to the bones (Bubendorf et al. 2000, Harada et al. 1992). CRC has many risk factors; the most important and well-evident ones are gender (males have an increased risk), age (direct relationship with the risk of developing CRC), family history, which increases the risk of developing inherited CRC syndromes such as hereditary nonpolyposis colorectal cancer (HNPCC) (that also referred to as Lynch syndrome) and familial adenomatous polyposis (FAP). Besides, patients having Inflammatory Bowel Disease (IBD) or previously diagnosed with CRC also have a higher risk of developing CRC (Cottet et al. 2012, Czene et al. 2002, Jess et al. 2012, Schoen et al. 2015). Approximately 20% of CRC patients already have metastasis at the time of diagnosis with the liver being the most common organ (Cejas et al. 2009, Sun et al. 2014, Pool et al. 2012).

Among the oldest chemicals used in medicine throughout history are the cannabinoids. These are chemicals derived from the *Cannabis sativa* plant and have been used for thousands of years for their medicinal purposes and their well-known strong psychotropic effects. The oldest reports that mention the medical use of *cannabis* go back to 2700 B.C in China and are mentioned in the Pen Ts'ao Ching (The Classic of Herbal Medicine) for the treatment of migraine, constipation, asthma, and malaria (Touw 1981). Besides, people in India grew cannabis and used it as preparations (Bhang) to reduce phlegm (Grierson 1894). The use of cannabis has been very controversial worldwide. It has been introduced into the United States Pharmacopoeia (USP) in 1851. It gained its popularity because other hypnotics and analgesics were not yet discovered, e.g., chloral hydrate was discovered in 1869, and paraldehyde and barbitals were identified in the following 30 years (Todd 1939). In 1942, however, cannabis was removed from the Pharmacopoeia due to its abuse potential, variation in its quality, fear of its unidentified active compounds, and because other conventional medications that were known for their efficacy were used as alternatives (Zuardi 2006). Many countries around the world allowed the use of cannabis for medicinal purposes and some countries made it legal partially or under certain conditions (Hammond et al. 2020).

Researchers found more than 500 compounds in cannabis, of which, 60 are considered phytocannabinoids (Hanuš et al. 2016). The most common and extensively researched cannabinoids (CBD), which differ in their structures are cannabinol (Wood et al. 1899), delta 9-tetrahydrocannabinol (Δ^9^-THC) (Gaoni and Mechoulam 1964), and cannabidiol (Mechoulam and Shvo 1963). CBD is a non-psychoactive chemical present in the cannabis plant (Ahmed 2022). It has been demonstrated that it possesses anti-inflammatory, analgesic, and anti-tumor activities (Ahmed 2022). CBD may be useful in combating chemotherapy resistance in cancer cells (Ahmed 2022). A study discovered that CBD may be able to sensitize canine cancer cells to chemotherapy treatments (Ahmed 2022). THC is the major cannabinoid causing the psychoactive effects of cannabis. On the other hand, the Endocannabinoid System ECS (Gorzo et al. 2022) endogenously produced by our body consists of endocannabinoids, cannabinoid receptors (CBs), and the enzymes that break down endocannabinoids after executing their functions. So far, researchers have discovered two endocannabinoids, N-arachidonoylethanolamine (AEA; also referred to as anandamide) in addition to 2-arachidonylglycerol (2-AG) (Kwee et al. 2023). Cannabinoid receptors namely cannabinoid receptor 1 (CB1) and cannabinoid receptor 2 (CB2) were discovered a few years later (Han et al. 2023). Recently, G protein-coupled receptors 55 and 119 (GPR55 and GPR119) were identified as cannabinoid receptors (dos Santos Sampaio MdF, de Paiva YB, Sampaio TB, Pereira MG, Coimbra NC. 2023) along with the transient receptor potential (TRP) channels which are a group of ion gated channels mostly located on the plasma membrane of several animal cell types (specifically TRPA1, TRPM8, TRPV1, and TRPV2) (Fernanda et al. 2023). CB1 receptors are heavily distributed in the Central Nervous System (CNS); predominantly occupying the basal ganglia and hippocampus (Yanar a, Karolin, Yazıcı Z. Cannabis 2023). In fact, CB1 receptors are considered the most copious type of receptors in the CNS (Araújo et al. 2023). While, CB2 receptors are expressed extensively in immune and hematopoietic cells, tonsils, and spleen, in addition to their presence in low quantities in the CNS (Brust et al. 2023). The major enzymes in the ECS responsible for degrading endocannabinoids are Fatty acid amide hydrolase (FAAH) that mainly works on AEA (Ramsay et al. 2023) and Monoacylglycerol Lipase (Han et al. 2023, Vatrella et al. 2020) that mainly degrades 2-AG (Simard et al. 2022).

**Fig. 1:**
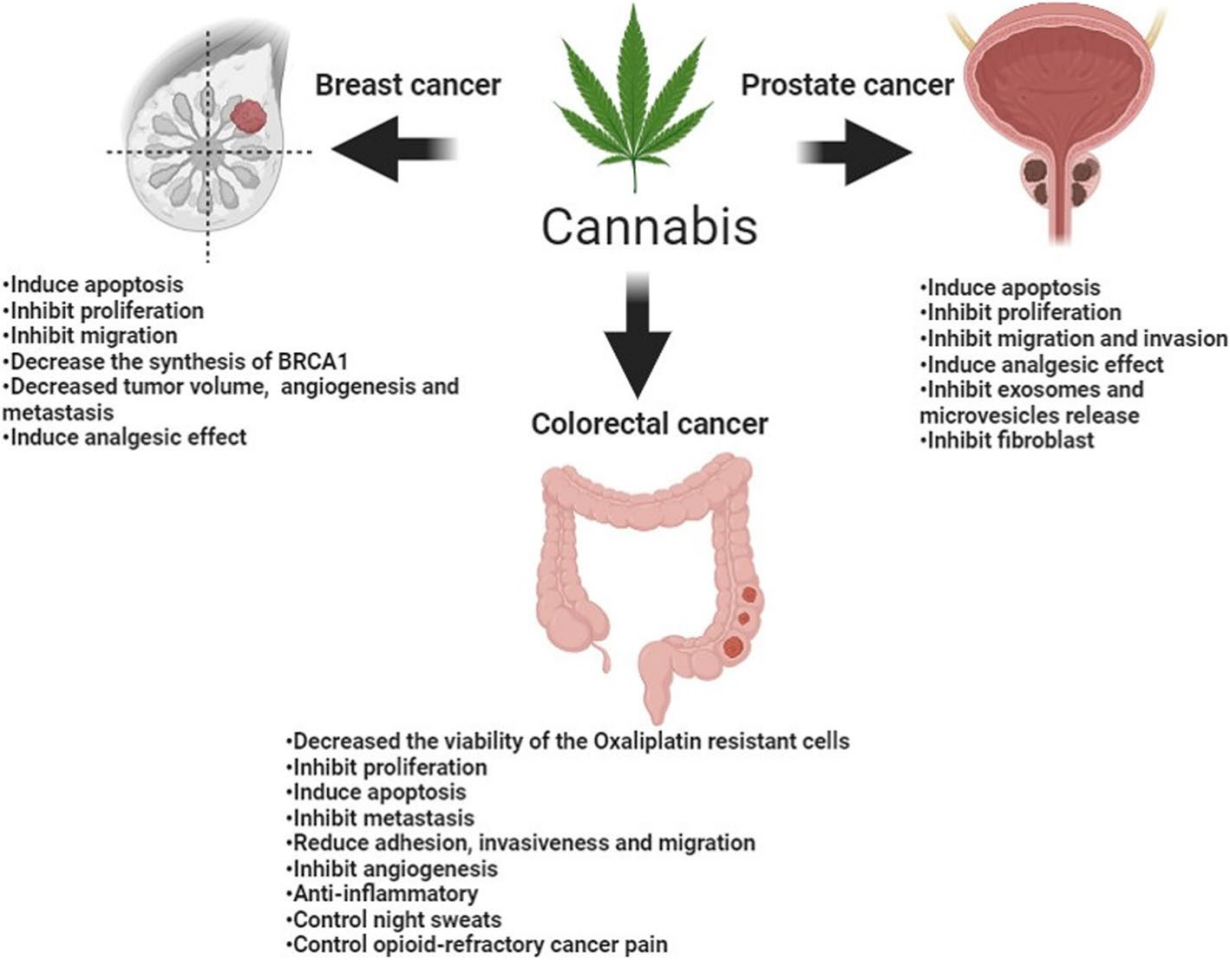
Summary of Cannabis effects on breast, colorectal, and prostate cancer

Recently, many studies have proven the expression of the different components of ECS in breast, colorectal, and prostate tumors. These studies demonstrate that medications targeting these components could be advantageous as antineoplastic agents. In this review, we will discuss this evidence in-depth and highlight any potential clinical values for cannabinoids in the three cancer types. Figure 1 summarizes the main effects of cannabinoids on breast, colorectal, and prostate cancer.

## Cannabis in breast cancer

BC is highly prevalent in women compared to men, as 10% of women develop BC at any point in their lives (Ammar-Shehada et al. 2023). BC has different pathological and molecular subtypes, and each of these subtypes has different treatments with each showing distinct outcomes (Furtney et al. 2023). Although some therapies showed great success in treating BC some subtypes of BC did not respond adequately to these therapies, and some of them relapse. Thus, the need for new therapies are emerging, and the real challenge is to find specific therapy that targets a specific subtype of BC (Bimonte et al. 2023).

Many risk factors could enhance the opportunity to develop BC, such as genetic factors, nulliparity, increase hormone levels, and a decrease in both iodine level and breastfeeding (Bimonte et al. 2023). As, the most common breast tissues from which cancer originates are the milk ducts (ductal carcinomas) and the lobules (lobular carcinomas), which secrete milk into ducts (Kadys et al. 2023). Preclinical evidence has arisen in the last recent years about the effectiveness of cannabinoids in treating cancer. This evidence has been tested both *in-vivo and in-vitro* on both cell cultures and mice (Voicu et al. 2023).

### Cannabinoid receptors in breast cancer

Different breast cancers have shown the expression of cannabinoid receptors (CB). In human breast carcinoma, the expression of CB1 immunoreactivity was 28% while for CB2 it was 35% (Prateeksha et al. 2023). A study found that CB1 receptor was expressed in 14% of the tumors and CB2 was expressed in 72% of the tumors, and in 91% of ErbB2-positive breast tumors, CB2 was expressed (Oliveira et al. 2023). This suggests that there is a link between CB2 and ErbB2- expression, but not between CB1 and ErbB2- expression (Selvaraj et al. 2023). ErbB2 (HER2) is a gene that encodes a protein involved in cell growth and division. In some subtypes of BC, ErbB2 is overexpressed or amplified, making it more aggressive and less susceptible to hormone therapy (Selvaraj et al. 2023). One of the BC subtypes that may be detected by measuring the expression of ErbB2 is ErbB2-positive BC (Selvaraj et al. 2023). ErbB2's oncogenic effect is mediated by a complex signaling network that closely regulates malignant cell motility and invasion, and therefore metastatic potential (Selvaraj et al. 2023). Recent attempts have been undertaken to discover gene expression profiles of ErbB2-positive invasive breast tumors, which may be key mediators of ErbB2-induced tumorigenesis and metastatic development (Selvaraj et al. 2023).

GPR55 is a new cannabinoid receptor, and a study found that its expression is extensive in the highly metastatic MDA-MB-231 cell lines and low by 30 folds in the low metastatic MCF-7 cell line (Morales et al. 2023). This study revealed that stimulating GPR55 by its endogenous ligand L-α lysophosphatidylinositol (LPI) stimulates cell migration and invasion in an MDA-MB-231 cell line. When the MCF-7 cell line transfected with pcDNA3.1 plasmid encoding human HA-GPR55 to increase the expression of GPR55, LPI enhanced the migration of the MCF-7 cell line. When the authors pretreated the MDA-MB-231 cell line with cannabidiol (which acts as a GPR55 antagonist, the effect of LPI on migration significantly decreased (Morales et al. 2023). On the other hand, another study reported that LPI enhances cell proliferation and Cannabidiol (CBD) blocked this effect (Martínez-Aguilar et al. 2023).

### Anticancer effects of cannabinoids against breast cancer

The effect of cannabinoids on different breast carcinoma cell lines have been examined in-*vitro,* and confirmed their capability to enhance apoptosis and inhibit both proliferation and migration (Lin et al. 2023). A study found that ∆^9^-THC induces apoptosis and inhibits BC cell proliferation through the activation of CB2 receptor (Zhong et al. 2023). A metabolite of ∆^9^-THC called Cannabinol (CBN), which acts as an agonist on CB1 and CB2 receptors found to inhibit cell proliferation (Bimonte et al. 2023). As well, it was stated that endogenous CB1 agonists as Anandamide, oleamide, and 2-Arachidonoylglycerol (works as CB1 and CB2 agonist) have an antiproliferative effect (Coelho et al. 2023). Moreover, arvanil (which is a synthetic CB1 agonist) also inhibits cell proliferation (Tundidor et al. 2023). Phytocannabinoids compounds were also tested in a study to investigate their abilities in inhibiting BC growth. The study found that CBD was the most potent, followed by Cannabigerol, then cannabichromene, while cannabidiol acid was the weakest compound (Younes 2023).

Moreover, a research has shown an inhibitory effect for exosome and microvesicle (EMV), which play a crucial role in tumor metastasis and released by several cancer types including BC (Tomko et al. 2022). Exosomes are defined as the smallest vesicles (30–100 nm) fused to the plasma membrane of the cell and releasing multivesicular bodies to the out-side milieu (Tomko et al. 2022). Microvesicles are vesicles (0.1–1.0 μm) that are produced from cells and shed by outward blebbing of the cell membrane (Tomko et al. 2022). CBD enhanced cisplatin apoptotic effect against MDA-MB-231 cancer cells, in addition to significantly reducing cell viability when cells were pretreated with CBD compared to the use of cisplatin alone (Kosgodage et al. 2018). Cisplatin is an alkylating agent, which is a kind of chemotherapy medication (Dasari et al. 2022). Platinum is present in it. It causes irreversible harm to the DNA of dividing cells (Dasari et al. 2022). This causes cancer cells and other quickly dividing cells to die by stopping or slowing their proliferation (Dasari et al. 2022). Cisplatin is licensed to treat bladder cancer, ovarian cancer that has spread to other parts of the body, and testicular cancer that has spread to other parts of the body alone or in combination with other medications. It is also being researched as a therapy for other forms of cancer (Dasari et al. 2022). CBD is a non-psychoactive chemical present in the cannabis plant (Ahmed 2022). It has been demonstrated that it possesses anti-inflammatory, analgesic, and anti-tumor activities (Ahmed 2022). CBD may be useful in combating chemotherapy resistance in cancer cells (Ahmed 2022). A study discovered that CBD may be able to sensitize canine cancer cells to chemotherapy treatments (Ahmed 2022).

Anandamide was found to decrease the synthesis of prolactin receptors, which reduces the effect of prolactin on breast cells. This leads to decrease the synthesis of BC susceptibility gene product (BRCA1), which is a marker for the proliferation of human mammary epithelial cells (Custódio et al. 2022). However, ∆^9^-THC decreases the proliferation of BC cells by arresting the cell cycle at G2-M, which leads to the induction of apoptosis. This effect was explained through activating CB2 receptors and subsequently reducing cyclin-dependent kinase-2 (Cdc2) levels, which makes the cell enter mitosis (Feng et al. 2022). This leads to cell cycle arrest and subsequent apoptosis (Zhong et al. 2023). Study found that cannabinoid receptor agonists, JWH-133 and WIN-55,212-2, decreased cell viability and migration in BC cell lines, MDA-MB-231, and MDA-MB-468 cell lines (Khunluck et al. 2022). It also found that JWH-133 and WIN-55,212-2 decreased tumor volume and angiogenesis in a group of mice that were subcutaneously injected with BC cell line, MDA-MB-231 cells. It also found that both JWH-133 and WIN-55,212 reduced lung metastasis (Khunluck et al. 2022).

During another *in-vivo* study, a group of MMTV (mouse mammary tumor virus)-neu mice (mice with BC) was divided into three experimental groups: control group (*n*=15), 6 received THC treatment, and 8 received JWH-133, which is a synthetic CB2 receptor-selective agonist (Caffarel et al. 2010). At the end of treatment, the THC and JWH-133 groups showed slower tumor growth, and the lesion was smaller than the control group. Also, the number of tumors developed in the cannabinoid-treated mice was less than three tumors, while 41% of the control group showed four or more than four tumors. The researchers discovered that THC and JWH-133 caused apoptosis (shown by an increase in the number of active caspase 3-positive cells), inhibited tumor cell proliferation (as demonstrated by a decrease in the number of Ki67 positive cells), and had an anti-angiogenic effect by reducing the number of blood vessels and vascularization (as shown by CD 31 staining). Additionally, they discovered that THC reduced the proportion of animals with lung metastasis in comparison to the control group, which displayed 67% of the mice to have lung metastasis. The JWH-133 group did not exhibit a decrease in this proportion, but 50% of the lesions were smaller and could only be seen under a microscope (Caffarel et al. 2010).

Furthermore, the phytocannabinoid cannabidiol also reduces tumor growth and decreases the number of lung metastases in mice injected with 4T1 BC cell lines (Suttithumsatid et al. 2023). Moreover, the Anandamide analogue, Methanandamide, also reduces the number and size of lung tumor nodules in mice injected with TSA-1 mammary carcinoma cell line through a CB1 receptor mechanism . Strikingly, when the CB1 antagonist SR141716A administered alone, has also been reported to decrease tumor size in mice injected with MDA-MB-231 cancer cells (Li et al. 2022).

### Effects of cannabinoids on COX-2 and prostaglandins in breast cancer

**Fig. 2:**
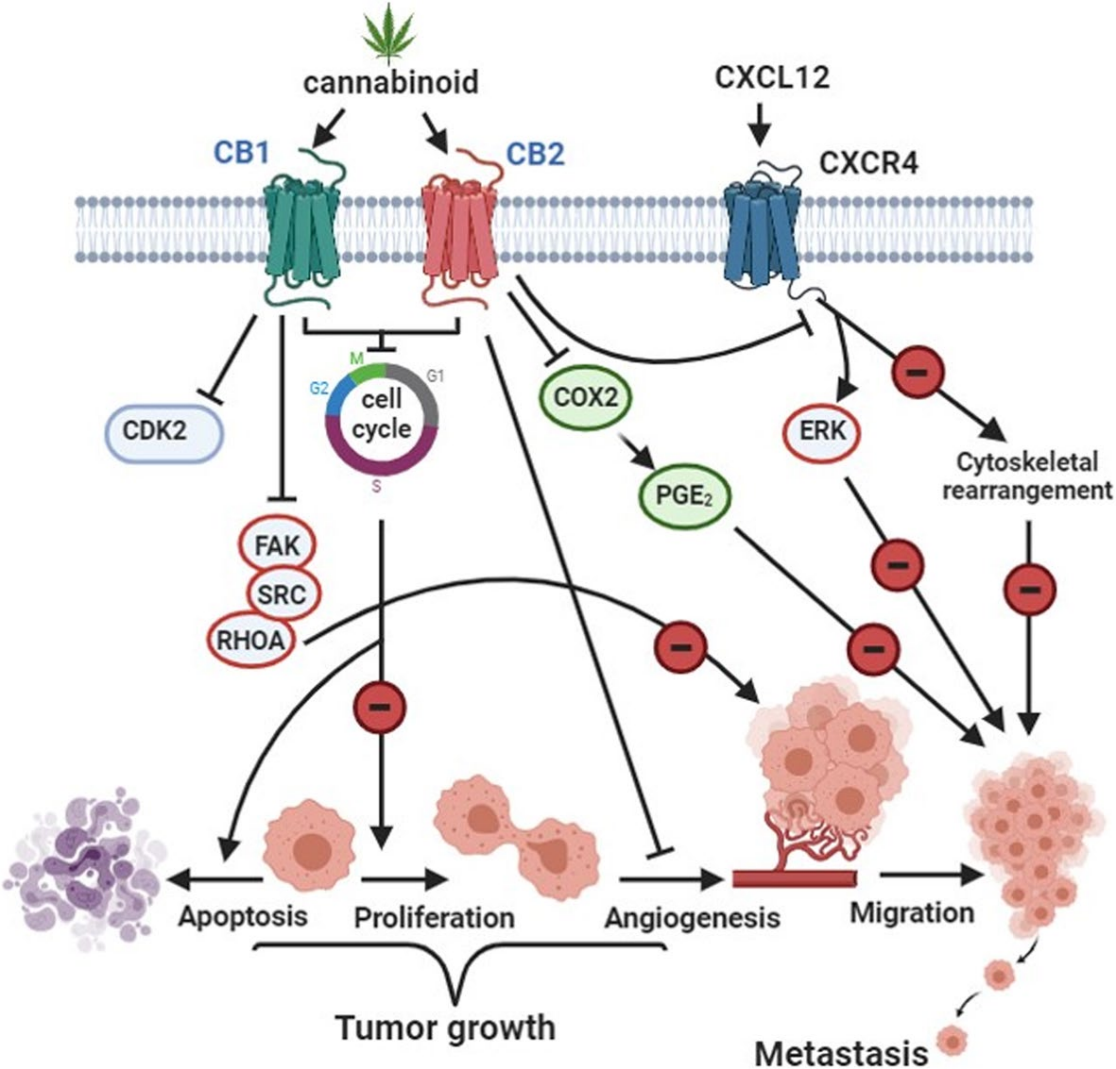
Cannabinoid effect on BC. This figure has been redrawn form (Caffarel et al. 2012)

In about 40% of breast cancers, there is overexpression of Cyclooxygenase-2 (COX-2). COX-2 produces prostaglandin-E2 (PGE_2_), which promotes angiogenesis and tumor growth (Sahu et al. 2023). Treating MDA-MB-231 cells with JWH-133 or WIN-55 resulted in decreasing the levels of PGE_2_ in the supernatants of MDA-MB-231 cells compared to the control group. There was also a decrease in (COX-2) expression in JWH-133– and WIN-55,212-2–treated MDA-MB-231 cells (Shah et al. 2021). As shown in Fig. 2, reducing PGE2 and COX-2 levels prevent cancer cell migration and metastasis.

## Cannabis in colorectal cancer

CRC is one of the most worldwide spread of cancers. In 2018, the world health organization declared that CRC is the third most common cancer diagnosed in the world by 1.8 million cases and the second cause of death from cancer worldwide by 862,000 deaths (Wang et al. 2023). It Is the third common cancer in men after lung and prostate cancer and the second common cancer in women after BC.

CRC arises more common in the sigmoid part of the large intestine and the rectum (Ingleby et al. 2022). The causes and risk factors of CRC are multiple. Some of these risk factors are Inflammatory bowel disease, family history, obesity, diet, lifestyle, age, smoking, and genes (Hossain et al. 2022). Most colorectal cancers are preceded by adenomas and polyps (i.e. precancerous lesions) (Garber and Chung 2022).

The current treatment of CRC is the surgical removal of the tumor followed by chemotherapy such as Oxaliplatin, Fluorouracil, and Leucovorin (Garber and Chung 2022). Unfortunately, studies have shown that patients develop resistance against some chemotherapies such as Oxaliplatin and Fluorouracil (Hsieh et al. 2022). Accordingly, there is a serious need for new therapies to treat CRC and to overcome this resistance.

Cannabis has shown good evidence to be effective in CRC at both levels *in-vivo* and *in-vitro*. It shows anticancer effects either alone or in combination with other chemotherapies (Perera and Diddeniya 2022). However, most of the in-*vivo* trials were conducted on animal models. Therefore, further clinical trials on humans are required to confirm its clinical effectiveness and safety.

### Cannabidiol in chemotherapy-resistant colorectal cancer

Oxaliplatin is a chemotherapeutic medication that is used to treat cancer. It is a platinum medication with alkylating properties (O'Dowd et al. 2023). Oxaliplatin, like other alkylating drugs, operates by interfering with the development of DNA in a cell. It kills cells by preventing them from growing and replicating (O'Dowd et al. 2023). This aids in the treatment of cancer, which is characterized by uncontrollable cell growth and division (O'Dowd et al. 2023). Exploring novel techniques to improve the efficacy of CRC treatment by identifying molecules and mechanisms linked with oxaliplatin resistance is necessary (Jeong et al. 2019). CBDhas the potential to assist human CRC cells overcome Oxaliplatin resistance. Jeong et al. conducted a study to demonstrate the effect of CBD on inducing autophagy in Oxaliplatin resistance colorectal cancer cell (CRC), they generated oxaliplatin-resistant cell lines, which didn’t respond to oxaliplatin treatment (Jeong et al. 2019). When the cell lines were treated with a combination of CBD and oxaliplatin, the death of oxaliplatin-resistant CRC was considerably raised (Jeong et al. 2019). The authors also performed an *in-vivo* study on mice. They injected a group of mice with oxaliplatin-resistant cell lines subcutaneously, then they measured the tumor size and weight every 2 days. They found that both size and weight of tumor were lower in mice that were treated with both oxaliplatin and CBD than in the non-treated control group and mice that were treated with either drug. The mechanism behind this is that CBDdecreases NOS_3_ phosphorylation-which is essential for Oxaliplatin resistance development- and superoxide dismutase-2 (which is an intracellular antioxidant) increasing Reactive Oxygen Species (ROS) through mitochondrial dysfunction leading to induce autophagy (Jeong et al. 2019).

Autophagy (macroautophagy) is a lysosomal breakdown of cytosolic proteins, damaged organelles, and invasive microorganisms in autophagosomes, which are double-membrane vesicles formed by phagophores extending (Jeong et al. 2019). Chemotherapy causes stress in cells, increasing apoptosis inhibition, autophagy, and EMT-competent phenotypes via Beclin-1, Bcl-2, mammalian target of rapamycin (mTOR), adenisine monophosphate (AMP)-activated protein kinase (AMPK), and select microRNAs (Jeong et al. 2019). CBD promotes oxaliplatin-mediated autophagy via NOS_3_ -mediated mitochondrial dysfunction, implying that NOS_3_ is a viable therapeutic target for overcoming oxaliplatin resistance and that CBD could be a novel treatment option for CRC (Jeong et al. 2019).

### Anticancer effects of CBD against colorectal cancer

#### Effect on apoptosis

A recent study was conducted both in *vivo* and in *vitro* to demonstrate the mechanism of CBD in inducing apoptosis in colorectal cancer cells. CBD reduced the viability of colorectal cancer cells by causing apoptosis, as evidenced by increased production of apoptotic markers. The authors discovered that CBD activated Noxa (a protein associated with apoptosis (Jeong et al. 2019) and that Noxa activation increased ROS generation, resulting in DNA damage and apoptosis (Jeong et al. 2019).

CBD triggered apoptosis via regulating numerous pro- and anti-apoptotic proteins, of which Noxa showed significantly higher expression (Jeong et al. 2019). To further understand the link between Noxa and CBD-induced apoptosis, Noxa levels were reduced using siRNA, and the expression of apoptotic markers was reduced (Jeong et al. 2019). After ROS production was inhibited, the level of Noxa fell, suggesting that ROS is involved in the control of Noxa, which is a well-known pro-apoptotic signaling agent along with ROS (Jeong et al. 2019). As a consequence, in a Noxa- and ROS-dependent way, CBD promoted apoptosis (Jeong et al. 2019).

Another mechanism by which CBD induces apoptosis is increasing the expression of death receptor-5 (DR5), to which TNF-Related Apoptosis-Inducing Ligand (TRAIL) binds and stimulates apoptosis in colorectal cancer cells, which in return increases the sensitivity to TRAIL leading to increased apoptosis in CRC, but it didn’t affect the normal colorectal cells .

#### Effect on metastasis

Metastasis, especially liver metastasis, is the reason behind the poor diagnosis of most CRC (Dillekås et al. 2019). It is the most common cause of death in CRC patients. Early detection and treatment of CRC have a 90% five-year survival rate, but once metastasis occurs this rate decreases to 10 to 15% (Cherkasova et al. 2021). A population-based study on CRC liver metastases found that 25-30% of patients with CRC have liver metastases, which is a primary cause of cancer-related fatalities (Dillekås et al. 2019). The incidence rate of metastasis that results in mortality in CRC patients, on the other hand, is not expressly reported (Dillekås et al. 2019).

To treat CRC metastasis, CBD can be used. CBD acts as an antagonist to GPR55 (Pulgar et al. 2022). GPR55 activation has been demonstrated to promote cancer metastasis via the G12/13 proteins (Rasheed et al. 2022). A study discovered that after *in-vitro* treatment of colorectal cancer cells with CBD, CBD inhibited the GPR55 receptor, lowering colorectal cancer cell adhesion, invasiveness, and migration (Wang et al. 2023).

#### Effect on proliferation

Another mechanism by which CBD fights CRC is through the suppression of cell proliferation. Aviello et al. tested the ability of CBD to decrease colorectal cancer cells’ proliferations. CBD was demonstrated to have a substantial antiproliferative affect (Aviello et al. 2012). They also conducted an *in-vivo* trial on mice to examine cannabidiol's ability as chemo-preventive therapy (Aviello et al. 2012). CBD was investigated for its ability to inhibit the production of aberrant crypt foci (ACF), polyps (both of which are precancerous lesions) (Reddy et al. 2023), and tumors in mice treated with azoxymethane (AOM) (carcinogenic compound effective for the induction of a colon carcinoma) (Aviello et al. 2012). They discovered that when compared to the control group, CBD dramatically reduced the formation of ACF, polyps, and tumors in cannabidiol-pretreated mice (Aviello et al. 2012).

#### Effect on angiogenesis

Angiogenesis is very important for cancer to progress and metastasize which makes this process an effective target for cancer treatment (Praphasawat et al. 2023). Honarmand et al. found that CBD has an anti-angiogenesis effect subsequently, has an anticancer and antimetastatic effect on a group of mice with colon cancer by reducing the expression of VEGF (vascular endothelial growth factor) in cannabidiol-treated mice more than the non-cannabidiol-treated control group (Honarmand et al. 2019). The same study also reported a decrease in the tumor size in a cannabidiol-treated group compared with the non-cannabidiol-treated group (Honarmand et al. 2019)).

### Anti-inflammatory effect of cannabinoids in colorectal cancer

Inflammation can cause cancer and vice versa (Taffoni et al. 2023). Cannabinoids showed promising results in treating both inflammations (which could cause cancer, such as ulcerative colitis and other inflammatory bowel diseases) and cancer-induced inflammation (Bereketoğlu 2020**).** Study found that CB1 and CB2 agonists administration reduced the mice-colon inflammation, cellular infiltration subsided and the epithelium returned to the normal appearance (Wardill et al. 2023). The effect of endocannabinoid system agonists on inflammatory bowel disease was reviewed deeply for 51 publications (Nduma et al. 2023). Scientists found that cannabinoids significantly reduced macroscopic colitis severity, expressed by disease activity index (DAI) (Nduma et al. 2023).

Besides the anti-inflammatory effect prior to cancer, cannabinoids also decrease cancer-induced inflammation (Lyons 2023). Honarmand et al. found that inflammatory cytokines, IL-6 and IL-8, in mice with colon cancer were elevated significantly in the serum compared to normal mice; indicating the presence of cancer-induced inflammation. Moreover, in cannabidiol-treated mice, the IL-6 and IL-8 serum levels were significantly lower than the untreated mice with colon cancer (Honarmand et al. 2019), indicating that CBD decreases the cancer-induced inflammation (Honarmand et al. 2019).

### Controversy

Although all previous evidence about the effectiveness of cannabis in treating CRC, there is a controversy about its anti-cancer effect. Some studies have shown that CB2 receptor activation with small-dose of exogenous agonist, induces cell proliferation leading to CRC (Lee et al. 2022). This study suggests that a low-dose agonist similar to the dose of endogenous cannabinoids promotes cancer progression. On the contrary, large doses of this agonist inhibit cancer development. Accordingly, physiological doses of CB2-agonists could promote cancer development, but using pharmacological doses (high) will inhibit cancer growth. Further studies must be conducted to clarify the effect of each receptor on CRC and to test the dose-dependent effect of cannabinoids on CB2 receptor.

## Cannabis in prostate cancer

PC has been reported as the fourth most common cause of cancer globally. World health organization (WHO) estimated the prevalence of PC in 2018 as 1.3 million cases worldwide (Pak et al. 2022). PC comes in second place as the most common cancer in men (13.5% of total cancer cases in men) after lung cancer (14.5%) (Maldonado Ortiz 2022).

Current treatments of local non-metastatic PC are active surveillance, radical radiotherapy, or radical proctectomy (Reddy et al. 2023). In metastatic PC, androgen-deprivation therapy (ADT) is used, and the castration is accomplished either by surgical, chemical with anti-androgens, luteinizing hormone-releasing hormone (LHRH) agonists, or antagonists (Zhang et al. 2023). Unfortunately, after 2 to 3 years, resistance starts to develop in PC against ADT, which is then become castration-resistant prostate cancer (CRPC) (Zhang et al. 2023). Several drugs have been used to overcome this resistance such as docetaxel, abiraterone acetate, cabazitaxel, enzalutamide, taxanes, radium-223, and sipuleucel-T (Dell’Atti and Aguiari 2023). All the previous drugs have demonstrated relatively small survival benefits and resistance developed eventually (Zhang et al. 2023). So, still more new treatments are required.

Noteworthy, it has been demonstrated that prostatic cancer highly expresses cannabinoid receptors, CB1, and CB2. This expression is also associated with the severity of cancer; higher in the more aggressive cancers (Mahmoud et al. 2023). Cannabinoids showed good evidence for possible anticancer effects, through multiple mechanisms in inhibiting the growth and progression of PC.

The study found that treating prostate cancer cell lines with endocannabinoids resulted in a substantial drop in cell viability and an increase in the frequency of apoptotic cells. These findings were linked to an increase in the active form of caspase-3 and a reduction in Bcl-2, indicating apoptotic pathway activation. They also boosted the amount of Erk while decreasing the level of Akt. All of the above processes explain the decrease in cell viability and suggest that endocannabinoids may be beneficial in treating prostate tumors that do not respond to standard therapies (Singh et al. 2021). Caspase-3 belongs to the caspase family, which consists of 13 aspartate-specific cysteine proteases that play an important role in the execution of the apoptotic program (Asadi et al. 2022). It is mainly responsible for the cleavage of PARP during cell death (Asadi et al. 2022). Caspase-3 reduces ROS generation after activation by caspase-9 and is needed for the successful execution of apoptosis (Asadi et al. 2022). Activated caspase-3 cleaves a wide range of downstream substrates during apoptosis, resulting in the usual morphological alterations seen in apoptotic cells (Asadi et al. 2022). Bcl-2 is a member of a family of proteins that work together to decide a cell's destiny (Zupo et al. 2009). By maintaining the integrity of the mitochondrial membrane and inhibiting the release of apoptogenic chemicals, Bcl-2 suppresses apoptosis (Zupo et al. 2009). One important signaling cascade that controls a number of biological functions, including as cell division, proliferation, motility, and survival, is the extracellular signal-regulated kinase ERK pathway (Hirashima et al. 2023). Tumor cells that are exposed to different anticancer treatments may undergo apoptosis due to ERK activation (Hirashima et al. 2023).

A recent study was conducted both *in-vitro* and *in-vivo* experiments to investigate the effect of a synthetic cannabinoid, WIN-55, on PC. The authors conducted the *in-vitro* experiment using prostate cancer cell lines (PC3, LNCaP, and DU145). WIN-55 showed a dose-dependent antiproliferative effect on all three cell lines. The effect ranges from 46% to 69% reduction in cell proliferation according to the dose of WIN-55, and the cell line. Also, WIN showed a significant increase in the apoptotic cells’ viability in PC3 and DU145 cells in a dose-dependent manner. WIN also showed a significant increase in the percentage of cells in the G1 phase and a decrease in the percentage of cells in the S phase suggesting that WIN cause cell cycle arrest in prostate cancer cells, although a previous study reported that there was no significant difference in the distribution of PC3 cells treated with endocannabinoids across the cell cycle phases compared with the control non-treated group of PC3 cells (Singh et al. 2021). Also, they found that WIN significantly reduces the migration and invasion of prostate cancer cells. They did the *in vivo* experiment by injecting PC3 cells subcutaneously in mice. Then, they divided the mice into the control group and WIN-treated group. The WIN-treated group showed significant reductions in the tumor size compared to the control group (Pennant and Hinton 2023).

### Effect of cannabinoids on exosomes and microvesicles released in prostate cancer

Microvesicles may carry caspase-3 away from cells as a protective mechanism against apoptosis. Exosomes and microvesicles (EMV) are associated with tumor spread and chemotherapy resistance, through expelling drugs outside the cancer cells (Liu and Wang 2023). Their inhibition will enhance the accumulation of chemotherapy drugs inside the cancer cells and produce the same efficacy with the lower dose, accordingly potentiate the apoptosis in the cancer cells (Liguori and Kralj-Iglič 2023).

In general, cannabinoids have shown a significant inhibitory effect on EMV released by different types of cancer. A study revealed that PC3 cells produced much more EMVs than BC cells or hepatocellular cancer cells, and treatment of PC3 cells with CBD (using 1 and 5 μM) reduced the EMVs by 44.5% 98.1%, respectively (Kosgodage et al. 2018). The reduction in EMVs with 5 μM CBD was significantly greater than the reduction with Cl-amidine, which is an effective EMVs inhibitor used as a comparator intervention. The proposed mechanism for this substantial activity is a reduction in ATP production and proton leakage, in addition to the suppression of mitochondrial respiration resulting in absence of pseudo-apoptotic responses in the PC cancer cells (Kosgodage et al. 2018).

In parallel, CBD also significantly decreased EMVs release from the other tumor cell lines, human hepatocellular carcinoma (HEPG2 and ECACC) and human breast adenocarcinoma (MDA-MB-231) compared to their control cells (Kosgodage et al. 2018). Also, CBD enhanced the apoptotic effect of cisplatin on MDA-MB-231 and HEPG2 cancer cells (Kosgodage et al. 2018). Prior treatment with CBD then treatment with cisplatin significantly decreased cell viability more than cisplatin-treated cells without prior treatment with CBD (Kosgodage et al. 2018). This shows that CBD increases the sensitivity of cancer cells to chemotherapy drugs.

### Effect of Cannabinoids on cancer-associated fibroblasts in prostate cancer

Fibroblasts are the major components of the stroma of PC (Pederzoli et al. 2023). Fibroblasts are necessary for cancer progression, cancer metastasis, and being androgen-independent (Lasorsa et al. 2023). A recent study was conducted on three prostate cancer cell lines (PC-3 and DU-145, and LNCaP) and healthy prostate cells as control (PNT-1) (Pietrovito et al. 2020). Without harming healthy tissues, they discovered that WIN-55 can specifically reduce the cell viability of prostate cancer cell lines (Pietrovito et al. 2020). They found that there is a significant increase in the expression of CB1 and CB2 receptors in cancer-associated fibroblasts (CAFs) compared to the normal fibroblasts (HFPs), although treating the HFPs and CAFs with CBD showed more decrease in the viability than WIN-55, this indicates that CBD acts through different receptors, such as peroxisome proliferator-activated receptor (PPAR)-γ (Pietrovito et al. 2020). CAFs were associated with a significant decrease in the expression of α-smooth muscle actin, matrix metalloproteinase, and invasion abilities with WIN-55 treatment (Pietrovito et al. 2020).When PC-3 cells were incubated with CAFs treated with/out cannabinoids, WIN was able to significantly reduce the CAFs-induced invasion of PC-3 cells (Pietrovito et al. 2020).

In contrast, the same study (Pietrovito et al. 2020) also reported that endocannabinoid antagonists could inhibit both PC-3 cell migration and CAFs activity, which indicates that endocannabinoids can promote cancer progression (Pietrovito et al. 2020).

### Effect of cannabinoids on ADT-treated patients

One of the reasons behind PC recurrence and progression in the ADT-treated patients is the differentiation of the prostate cancer cells into the neuroendocrine (NE)-like cells which correlates with tumor progression and poor prognosis (Bennett et al. 2023). This occurs in a hormone-deficient medium, which occurs in patients receiving ADT (Bennett et al. 2023). Using this principle, Morell et al. experimented to see the inhibitory effect of WIN on the differentiation of the prostate cancer cells into neuroendocrine-like cells (Morell et al. 2016). They found that WIN decreased the cell viability in both LNCaP and the NE differentiated cells, and when they incubated the LNCaP cell line with WIN, it showed a significant decrease in the NE markers in the resulting NE cells (106). The way that WIN inhibits PI3K/Akt causes AMPK to be activated, which in turn reduces NE differentiation (Morell et al. 2016). This is the mechanism underlying the inhibitory action. This study also found that cannabinoid receptors show a decrease during NE differentiation, and cannabinoid receptors have a tonic inhibitory effect on NE differentiation (Morell et al. 2016).

Treatment for ADT adverse effects may potentially benefit from cannabinoids (Mousa et al. 2020). According to a study, the majority of patients with advanced PC who had androgen-deprivation treatment reported feeling somewhat relieved from its side effects (Mousa et al. 2020). For example, pain, fatigue, sleeplessness, hot flashes, irritability, depression, headache, nausea, and vomiting are common adverse effects of androgen deprivation therapy (ADT) for individuals with PC (Mousa et al. 2020). Numerous research studies have demonstrated the potential effectiveness of cannabis in treating neuropathic pain, nausea, and vomiting (Mousa et al. 2020).

## Palliative actions of cannabinoids in cancer

### Night sweats

Dronabinol (a synthetic form of delta-9-tetrahydrocannabinol) was found to be an effective therapy for paraneoplastic night sweats in cancer patients (Carr et al. 2019). Five patients were considered if they had a cancer diagnosis and complained of night sweats that interfered with their quality of life. All decided to try oral dronabinol to alleviate their night sweats (Carr et al. 2019). There were two female patients and three male patients. Two had Acute Myeloblastic Leukemia, while the others had colon cancer, rectal cancer, and BC. Patients were at various phases of disease-modifying treatments. Three patients were given 5 mg at bedtime, while one was started on 5 mg three times a day (TID) and gradually raised to 10 mg TID due to worsening symptoms (Carr et al. 2019). One older patient was given 2.5 mg twice a day (BID) to begin with. Patients were examined one to four weeks after starting treatment at their next planned appointment. One patient noticed a decrease in the intensity of night sweats three days after starting treatment. Two patients experienced total relief from nocturnal sweats (Carr et al. 2019). The other three patients indicated that the severity of their night sweats had decreased, requiring them to change clothes just once or not at all during the night. Due to sedation, one patient ceased the dronabinol and noted that his night sweats reappeared (Carr et al. 2019). Once the patients' night sweats were resolved, other symptoms such as anxiety and fatigue improved, leading to an improvement in their overall quality of life. All five patients' symptoms resolved after one week of starting dronabinol (Carr et al. 2019).

## Pain

The cannabinoid receptors are highly abundant in the brain (Patthy et al. 2023). Normally, Pain begins when tissue is damaged, as damaged tissues release nerve growth factor, which activates mast cells. Mast cells degranulate and produce bradykinin, which activates nociceptors. The impulse is then carried through peripheral nerve fibers to the dorsal root ganglia, which merges with the spinal cord and travels to the brain (Boissoneault et al. 2023). The brain responds by releasing gamma-Aminobutyric acid (GABA) and other substances to inhibit pain and excitatory impulses (Boissoneault et al. 2023). Interestingly, CB2 receptor is found on mast cells and upon its activation by CB2 ligands and nonselective CB1/CB2 agonists, it inhibits cell degranulation . This leads to decreasing bradykinin release and nociceptor stimulation. Moreover, CB1 receptors were also found to have an analgesic effect by reducing nerve-C-fiber-driven post-discharge responses .

In reality, opioids are the most common drugs used to relieve cancer-associated pain (Dupoiron et al. 2022). Therefore, more than one-third of the patients receiving opioids, suffer from opioid-refractory cancer pain, in addition to opioids’ adverse effects (Diernberger et al. 2023). This spotlight the need to find new drugs to be used alone or as add-on therapy with opioids. Remarkably, cannabinoids showed promising results in treatment-refractory patients (Sá et al. 2023). A randomized, placebo-controlled trial tested the effect of Nabiximols (which is a new cannabinoid drug) as add-on therapy on opioid-refractory cancer patients. It showed that Nabiximols-treated patients reported significant analgesic effect and a decrease in pain score more than the placebo group (Sá et al. 2023).

Furthermore, a recent retrospective study collected 232 cancer patients (53 patients with gastrointestinal cancer) and divided them into 137 patients with THC+ and 95 patients without THC-. It found that the THC+ group showed a decrease in opioid use by 33% and those in THC- showed an increase in opioid use by 23%. Moreover, in THC- a group it is required to increase the opioid daily use by 63% to achieve the same pain relief as the THC+ group (Pawasarat et al. 2020). Also, an observational study included 2970 cancer patients of which 236 patients had CRC, more than 50% of patients reported high pain score (8-10 out of 10) before starting treatment, but after 6 months of using medical cannabis, less than 5% reported such high score (Bar-Lev Schleider et al. 2022). The same study also reported an improvement in nausea and vomiting associated with cancer (Bar-Lev Schleider et al. 2022).

## Conclusion

Cannabinoids are chemicals derived from the *Cannabis sativa* plant and have been used for their medicinal purposes, especially for their well-known strong psychotropic effects. There is growing evidence supporting the role of Cannabinoids in numerous pathological conditions, including their role in several cancer types such as breast, colorectal, and prostate cancer. Accordingly, cannabinoids could have a promising potential as adjunctive therapy for the treatment of these types of cancers.

## Data Availability

Not applicable.

## Abbreviations

ACF: Aberrant crypt foci
ADT: Androgen-deprivation therapy
AEA: N-arachidonoylethanolamine; anandamide
AG: Arachidonylglycerol
AMPK: Adenosine monophosphate-activated protein kinase
AOM: Azoxymethane
BC: Breast cancer
BID: Twice a day
BRCA: Breast cancer susceptibility protein
CAF: Cancer-associated fibroblasts
CB: Cannabinoids receptor
CBD: Cannabidiol
CBN: Cannabinol
CDAI: Crohn's Disease Activity Index
Cdc: Cyclin-dependent kinase
CNS: Central nervous system
COX: Cyclooxygenase
CRC: Colorectal cancer
CRPC: Castration-resistant prostate cancer
DAI: Disease activity index
DNA: Deoxyribonucleic acid
DR: Death receptor
ECS: Endocannabinoid system
EMV: Exosome and macrovesicle
FAAH: Fatty acid amide hydrolase
FAP: Familial adenomatous polyposis
GABA: Gamma-aminobutyric acid
GPR: G-protein-coupled receptor
HNPCC: Hereditary nonpolyposis colorectal cancer
IBD: Inflammatory Bowel Disease
IL: Interleukin
LHRH: Luteinizing hormone-releasing hormone
LPI: Lysophosphatidylinositol
MAGL: Monoacylglycerol Lipase
MMJ: Medical marijuana
MMTV: Mouse mammary tumor virus
MV: Macrovesicle
NE: Neuroendocrine
PC: Prostate cancer
PG: Prostaglandin
PPAR: Peroxisome proliferator-activated receptor
TGF: Transforming growth factor
THC: Tetrahydrocannabinol
TID: Three times a day
TRAIL: TNF-Related Apoptosis-Inducing Ligand
TRP: Transient receptor potential
USP: United States Pharmacopoeia
VEGF: Vascular endothelial growth factor
WHO: World health organization
